# Exploration of the immune microenvironment of breast cancer in large population cohorts

**DOI:** 10.3389/fendo.2022.955630

**Published:** 2022-08-15

**Authors:** Youyuan Deng, Jianguo Wang, Zhiya Hu, Yurong Cai, Yiping Xu, Ke Xu

**Affiliations:** ^1^ Department of General Surgery, Xiangtan Central Hospital, Xiangtan, China; ^2^ Department of Pharmacy, Third Hospital of Changsha, Changsha, China; ^3^ Department of Oncology, The First Affiliated Hospital of Chengdu Medical College, Chengdu, China; ^4^ Clinical Medical College, Chengdu Medical College, Chengdu, China; ^5^ Key Clinical Specialty of Sichuan Province, Chengdu, China

**Keywords:** breast cancer, immune, drug, prognosis, signature

## Abstract

Tumor immune microenvironment is associated with tumor progression. However, previous studies have not fully explored the breast cancer (BC) immune microenvironment. All the data analyzed in this study were obtained from the open-access database, including The Cancer Genome Atlas, Gene Expression Omnibus (TCGA), and cBioPortal databases. R software v4.0 and SPSS 13.0 were used to perform all the statistical analysis. Firstly, the clinical and expression profile information of TCGA, GSE20685, GSE20711, GSE48390, GSE58812, and METABRIC cohorts was collected. Then, 53 immune terms were quantified using the single-sample Gene Set Enrichment Analysis (ssGSEA) algorithm. A prognosis model based on HER2_Immune_PCA, IL12_score, IL13_score, IL4_score, and IR7_score was established, which showed great prognosis prediction efficiency in both training group and validation group. A nomogram was then established for a better clinical application. Clinical correlation showed that elderly BC patients might have a higher riskscore. Pathway enrichment analysis showed that the pathway of oxidative phosphorylation, E2F targets, hedgehog signaling, adipogenesis, DNA repair, glycolysis, heme metabolism, and mTORC1 signaling was activated in the high-risk group. Moreover, Tumor Immune Dysfunction and Exclusion and Genomics of Drug Sensitivity in Cancer analysis showed that low-risk patients might be more sensitive to PD-1 therapy, cisplatin, gemcitabine, paclitaxel, and sunitinib. Finally, four genes, *XCL1*, *XCL2*, *TNFRSF17*, and *IRF4*, were identified for risk group classification. In summary, our signature is a useful tool for the prognosis and prediction of the drug sensitivity of BC.

## Introduction

Breast cancer (BC) is a prevalent malignant tumor all over the world, especially in women (1). According to molecular characterizations, BC could be generally divided into four subtypes, namely, luminal A, luminal B, human epidermal growth factor receptor 2 (HER2), and triple-negative BC (TNBC) (2). For resectable BC, surgery combined with chemotherapy is still the first-line treatment option with a relatively satisfactory prognosis (3). However, despite significant advances in modern medical technology, the therapeutic effect of advanced BC is still not satisfactory. Therefore, it is essential to further explore the underlying biological mechanism of BC development.

The tumor microenvironment has recently been receiving increasing attention from researchers worldwide (4). As one of the key components of the tumor microenvironment, immune cells and corresponding immune status drastically affect the malignant biological behavior of cancer cells (5). Meanwhile, treatment response may be influenced by the immune status of the tumor. In general, it is believed that the immune system is instrumental in maintaining a balance between immunity and tumor development and can act both as a promoter and an inhibitor of tumor growth. For instance, Narayanan et al. found that the M1-polarized macrophage infiltration in the tumor microenvironment could contribute to better treatment outcomes and survival rate of MSI-H colon cancer patients, indicating the antitumor effect of M1-polarized macrophages (6). In patients with solid tumors, neutrophils are released from the bone marrow under strong pressure, and most of these are usually immature, which might lead to cancer progression (7). Germain et al. indicated that the existence of B cells in tertiary lymphoid structures might be associated with the protective immunity in patients with lung cancer (8). Meanwhile, chemokines can regulate the interaction between immune cells and other cells in tumor tissue, which is very important for immune cell migration, as well as effective antitumor immune response (9). Also, researchers gradually realize that the immune system may be an effective way to fight tumor cells. By influencing immune cells against tumors, immunotherapy activates the immune system and attacks the tumor (10). Immune checkpoint inhibitor therapy has obtained promising results in solid tumors, mainly including PD-1/PD-L1 and CTLA-4 therapy (11). Thus, the exploration of the tumor immune microenvironment of BC might help to identify underlying biomarkers associated with cancer progression and therapy choice.

With the development of sequencing technology and bioinformatics, the massive data generated from this could assist researchers to get a deeper understanding of disease mechanisms (12). In our study, we comprehensively collected the clinical and transcriptional profiling information of multiple independent BC cohorts. Next, 53 immune terms were quantified based on the single-sample Gene Set Enrichment Analysis (ssGSEA) algorithm. Based on these immune terms, a prognosis model based on HER2_Immune_PCA, IL12_score, IL13_score, IL4_score, and IR7_score showed a great prediction efficiency in training and validation cohorts. Pathway enrichment analysis was then performed to explore the underlying biological differences between high- and low-risk patients. Moreover, we found that low-risk patients might be more sensitive to PD-1 therapy, cisplatin, gemcitabine, paclitaxel, and sunitinib. Finally, four genes, *XCL1*, *XCL2*, *TNFRSF17*, and *IRF4*, were identified for risk group classification.

## Methods

### Data acquisition

The open-access transcriptional profile and clinical information was downloaded from The Cancer Genome Atlas (TCGA, https://portal.gdc.cancer.gov/), Gene Expression Omnibus (GEO, https://www.ncbi.nlm.nih.gov/gds/?term=), and cBioPortal databases (https://www.cbioportal.org/). In brief, the corresponding data of BC patients in TCGA were obtained from the TCGA-BRCA project, in original “TPM” form and clinical information was in “xml bcr” form. All these data were collated using the author’s R code. GSE20685 (Platform: GPL570, Affymetrix Human Genome U133 Plus 2.0 Array), GSE20711 (Platform: GPL570, Affymetrix Human Genome U133 Plus 2.0 Array), GSE48390 (Platform: GPL570, Affymetrix Human Genome U133 Plus 2.0 Array), and GSE58812 (Platform: GPL570, Affymetrix Human Genome U133 Plus 2.0 Array) provide the expression profile and corresponding prognosis information of BC patients, which were identified from the GEO database. The METABRIC cohort was identified from the cBioPortal database, which provides targeted sequencing and clinical information of 2,509 primary breast tumors with 548 matched normals. The patients with expression profiles and complete prognosis information were included in our study. The following steps were carried out for all data before analysis: (i) missing value completion, (ii) probe annotation, and (iii) data normalization. The sva package was used to eliminate inter-assay variability. Moreover, mutation data were downloaded from the TCGA website and TMB and MSI scores were calculated. Based on the one-class logistic regression machine learning (OCLR) machine-learning algorithm, tumor stemness index (mRNAsi) was calculated (13). The baseline information of the enrolled patients is shown in **Tables S1–S6**.

### Immune quantification

Immune terms quantification was performed to quantify the normalized enrichment score (NES) of 53 immune cells and immune response based on the ssGSEA algorithm (14). The reference gmt file was obtained from the previous study (15).

### Model construction and validation

Firstly, the NES value of 53 immune terms was combined with patient survival information. Then, univariate Cox regression analysis, LASSO regression, and multivariate Cox regression analysis were performed to identify prognosis-related immune terms (16). The prognosis model was constructed with the following formula: “Riskscore = β1μ1 + β2μ2 + β3μ3+ … + βNμN”. Here, “β,” “μ,” and “N” represent the coefficient, NES value, and the number of selected immune terms, respectively. The prediction efficiency of the model was evaluated using the Kaplan–Meier and ROC curve. The AUC value of more than 0.65 was considered to have good predictive capabilities.

### Nomogram and calibration curve

For a better application of our model in clinical settings, the riskscore and clinical features were combined for the nomogram plot using the rms package. The calibration curve was used to compare the predicted survival based on the nomogram with the actual survival. The decision curve analysis (DCA) was used to analyze the effect of clinical features on riskscore.

### Pathway enrichment

Pathway enrichment was conducted using the Gene Set Enrichment Analysis (GSEA) and ssGSEA (14). For the ssGSEA, the reference gene set was c5.all.v7.5.1.symbols.gmt and msigdb.v7.5.1.symbols.gmt. For the GSEA, the reference gene set was Hallmark gene set. Gene oncology (GO) and Kyoto Encyclopedia of Genes and Genomes (KEGG) analysis was performed using the ClusterProfiler package in R software (17).

### Immunotherapy and drug sensibility

Tumor Immune Dysfunction and Exclusion (TIDE) and submap algorithm were used to compare the difference in immunotherapy response rate between different groups (18). Drug sensitivity analysis was performed based on the Genomics of Drug Sensitivity in Cancer (GDSC) database (19). The pRRophetic package was used for the prediction process.

### Machine learning algorithm

LASSO logistic regression and support vector machine recursive feature elimination (SVM-RFE) algorithms were used to identify characteristic genes. LASSO regression algorithm is one of the methods of machine learning that can identify the smallest classification error *λ* to determine variables (20). SVM-RFE algorithm is a machine learning method based on the SVM, which can find the best variable by deleting the feature vector generated by the SVM (21).

### Statistical analysis

R software v4.0 and SPSS 13.0 were used to perform all the statistical analysis. *p*-value was two-sided and <0.05 was considered statistically significant. An independent sample *t*-test was used to compare continuous variables with normal distribution, and Spearman correlation analysis was used for continuous variables.

## Results

### Immune terms quantification

**Figure 1:**
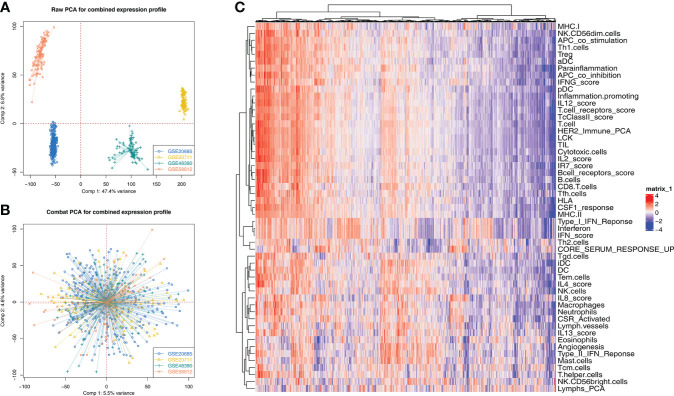
Data combination and immune terms quantification **(A)** Four GSE cohorts selected for our analysis have a significant batch difference, including GSE20685, GSE20711, GSE48390, and GSE58812; **(B)** The sva package used for cohort combination greatly reduces the batch difference. **(C)** ssGSEA algorithm was used to quantify 53 immune terms.

The whole flowchart of this study is shown in **Supplementary Figure 1**. GSE20685, GSE20711, GSE48390, and GSE58812 have the same platform, GPL570. Therefore, we try to merge these four independent cohorts into a total cohort. **Figure 1A** showed a significant batch effect between these cohorts (Comp 1: 47.4% variance, Comp 2: 6.8% variance). A remarkable decrease in the batch effect was observed after data combination using sva package (**Figure 1B**). Furthermore, ssGSEA algorithm was performed to quantify the 53 immune terms (**Figure 1C**).

### Prognosis model construction

**Figure 2:**
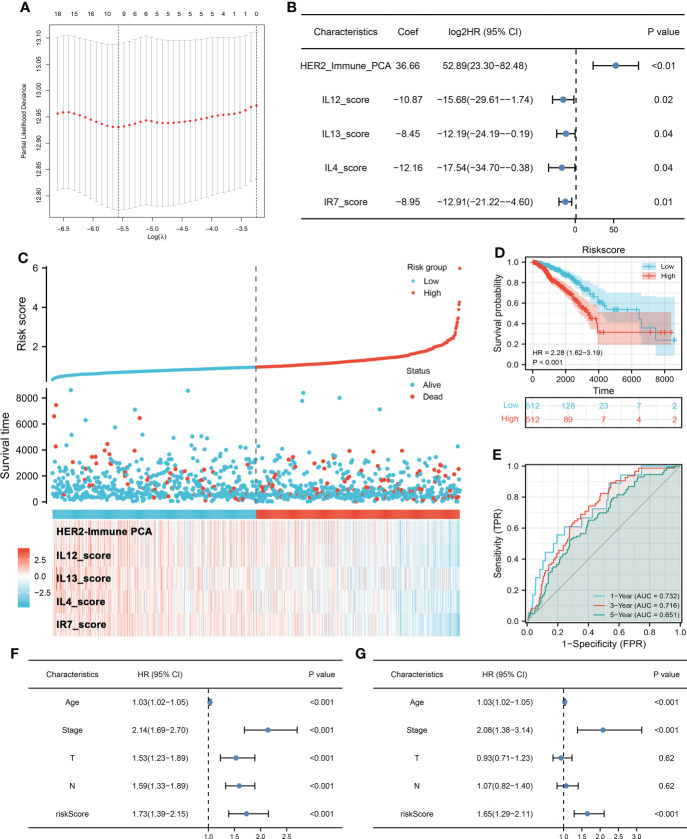
Prognosis model construction **(A)** LASSO regression was used for dimensionality reduction. **(B)** Multivariate Cox regression analysis was performed for model construction with the threshold of *p*-value < 0.05. **(C)** A prognosis model was finally established based on HER2_Immune_PCA, IL12_score, IL13_score, IL4_score, and IR7_score. **(D)** Kaplan–Meier survival curve showed that high-risk patients might have a worse prognosis. **(E)** ROC curve was used to evaluate the prediction efficiency of our model. **(F, G)** Univariate and multivariate Cox analysis indicated that riskscore is a risk factor independent of other clinical features.

Firstly, univariate Cox regression and LASSO analysis were performed to screen the prognosis-related immune terms based on the TCGA database (**Figure 2A**). The terms meeting the criteria of univariate Cox regression are shown in **Table S7**. Then, multivariate Cox regression analysis identified five immune terms used for model construction, namely, HER2_Immune_PCA, IL12_score, IL13_score, IL4_score, and IR7_score (**Figure 2B**). Correspondingly, each patient was assigned a riskscore with the formula of “HER2_Immune_PCA * 36.66 + IL12_score * −10.87 + IL13_score * −8.45 + IL4_score * −12.16 + IR7_score * −8.95”. All the patients were divided into high- and low-risk groups based on the median riskscore and more dead cases were observed in the high-risk group (**Figure 2C**). Kaplan–Meier survival curve showed that the patients in the high-risk group might have a worse prognosis (**Figure 2D**, HR = 2.28, *p* < 0.001). The ROC curve indicated that our model had a great prediction efficiency in patients OS (**Figure 2E**, 1-year AUC = 0.732, 3-year AUC = 0.716, 8-year AUC = 0.651). Furthermore, univariate and multivariate analysis showed that our model is an independent risk feature for BC patient prognosis (**Figures 2F, G**, univariate: HR = 1.73, *p* < 0.001; multivariate: HR = 1.65, *p* < 0.001).

### Model validation

**Figure 3:**
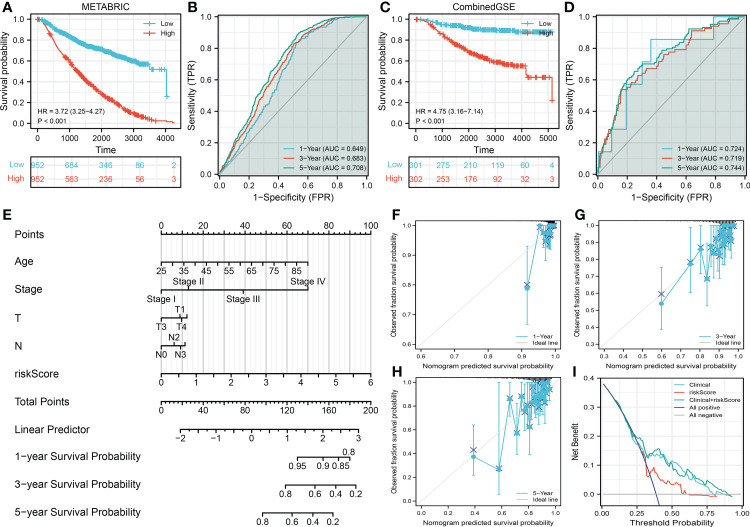
Validation of the prognosis model **(A, B)** Kaplan–Meier and ROC curves were performed to evaluate the prediction efficiency in the METABRIC database (validation cohort). **(C, D)** Kaplan–Meier and ROC curves were performed to evaluate the prediction efficiency in the CombinedGSE database (validation cohort). **(E)** A nomogram was established through combining the riskscore and clinical features for a better clinical application. **(F–H)** Calibration curve of 1-, 3-, and 5-year survival predicted by the nomogram. **(I)** Decision curve of the nomogram.

The METABRIC and CombinedGSE cohorts (GSE20685, GSE20711, GSE48390, and GSE58812) were used for the validation group. In the METABRIC group, the Kaplan–Meier survival curve also indicated that the high-risk patients tend to have a shorter OS (**Figure 3A**, HR = 3.72, *p* < 0.001). Meanwhile, we also found a good prediction efficiency in patient prognosis (**Figure 3B**, 1-year AUC = 0.649, 3-year AUC = 0.683, 5-year AUC = 0.708). Moreover, the same conclusions were also observed in the CombinedGSE cohort (**Figure 3C**, HR = 3.72, *p* < 0.001; **Figure 3D**, 1-year AUC = 0.649, 3-year AUC = 0.683, 5-year AUC = 0.708). Next, a nomogram was established for a better application in clinical with the combination of riskscore and clinical features (**Figure 3E**). Calibration curves showed a high fitting degree between the predicted survival based on the nomogram with the actual survival (**Figures 3F–H**). Also, the DCA curve showed that the nomogram is clinically useful (**Figure 3I**).

### Clinical correlation

**Figure 4:**
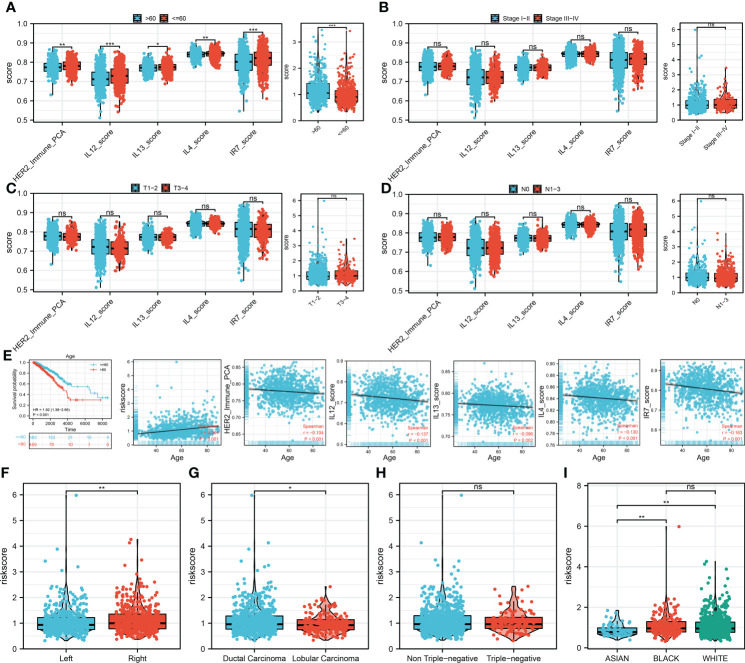
Clinical correlation analysis **(A)** The difference of model immune terms and riskscore in different age groups. **(B)** The difference of model immune terms and riskscore in different stage groups. **(C)** The difference of model immune terms and riskscore in different T stage groups. **(D)** The difference of model immune terms and riskscore in different N stage groups. **(E)** Elderly patients might have a worse prognosis compared with the younger patients. **(F–I)** The riskscore difference in different clinical groups. *P <0.05; **P < 0.01; ***P < 0.001.

We then performed the correlation analysis between clinical features and model immune terms, as well as the riskscore. Interestingly, the result showed that the older patients (>60) have a higher HER2_Immune_PCA, IL12_score, IL13_score, IL4_score, IR7_score, and riskscore level compared with younger patients (≤60) (**Figure 4A**). No significant difference was observed in the clinical stage, T, and N classifications (**Figures 4B–D**). Furthermore, we try to explore the prognosis difference between different age groups. The result showed that the prognosis is worse for older BC patients (**Figure 4E**). Correlation analysis indicated that the HER2_Immune_PCA, IL12_score, IL13_score, IL4_score, and IR7_score were negatively correlated with patient age, yet riskscore was positively correlated (**Figure 4E**). Interestingly, we found that the patients with right BC tend to have a higher riskscore (**Figure 4F**). Compared with the lobular carcinoma, the ducal carcinoma might have a higher riskscore (**Figure 4G**). No significant difference in riskscore was observed between triple-negative and non-triple-negative BC (**Figure 4H**). Of the different races, Asian populations might have the lowest riskscore (**Figure 4I**). We further explored the underlying biological differences between patients ≤60 years old and those >60 years old (**Supplementary Figure 2A**). The result showed that the pathways of fatty acid metabolism, early estrogen response, peroxisome, oxidative phosphorylation, late estrogen response, reactive oxygen species, androgen response, xenobiotic metabolism, heme metabolism, and adipogenesis were activated in high-risk patients.

### Pathway enrichment of our model

**Figure 5:**
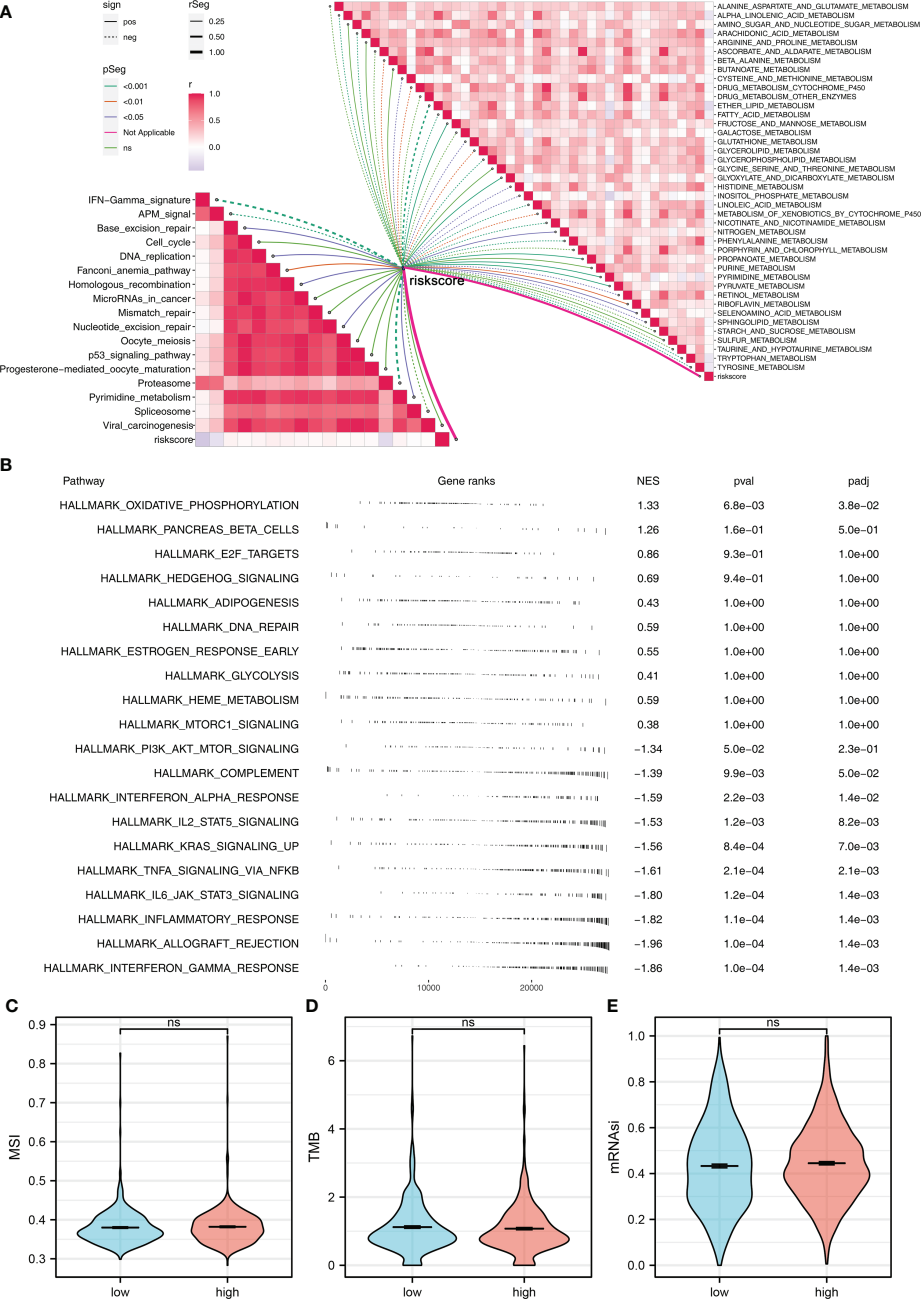
Pathway enrichment analysis **(A)** ssGSEA was performed to evaluate the correlation between riskscore and immune and metabolism pathways. **(B)** GSEA was performed to explore the underlying biological differences between high- and low-risk groups. **(C–E)** The TMB, MSI, and mRNAsi differences between high- and low-risk patients. ns P > 0.05.

To explore the underlying biological mechanism between high- and low-risk groups, we then performed pathway enrichment analysis. The result showed that in metabolism-related pathways, riskscore was positively correlated with pyruvate metabolism, glyoxylate and dicarboxylate metabolism, and fructose and mannose metabolism, but negatively correlated with ether lipid metabolism, alpha-linolenic acid metabolism, and tryptophan metabolism (**Figure 5A**). For immune-related pathways, riskscore was negatively correlated with IFN-γ signature, APM signal, and proteasome (**Figure 5A**). Based on the GSEA, the pathways of oxidative phosphorylation, E2F targets, hedgehog signaling, adipogenesis, DNA repair, glycolysis, heme metabolism, and mTORC1 signaling were activated in the high-risk group (**Figure 5B**). GO and KEGG analysis showed that the terms of nicotine addiction (hsa05033), transmitter-gated ion channel activity (GO:0022824), transmitter-gated channel activity (GO:0022835), extracellular ligand-gated ion channel activity (GO:0005230), calyx of Held (GO:0044305), ion channel complex (GO:0034702), transmembrane transporter complex (GO:1902495), and substrate-specific channel activity (GO:0022838) were significantly upregulated in high-risk patients (**Supplementary Figure 2B**). We further explored the differences in TMB, MSI, and mRNAsi between high- and low-risk patients. However, no significant difference was observed (**Figures 5C–E**).

### Riskscore effectively predicts the immunotherapy and chemotherapy sensitivity

**Figure 6:**
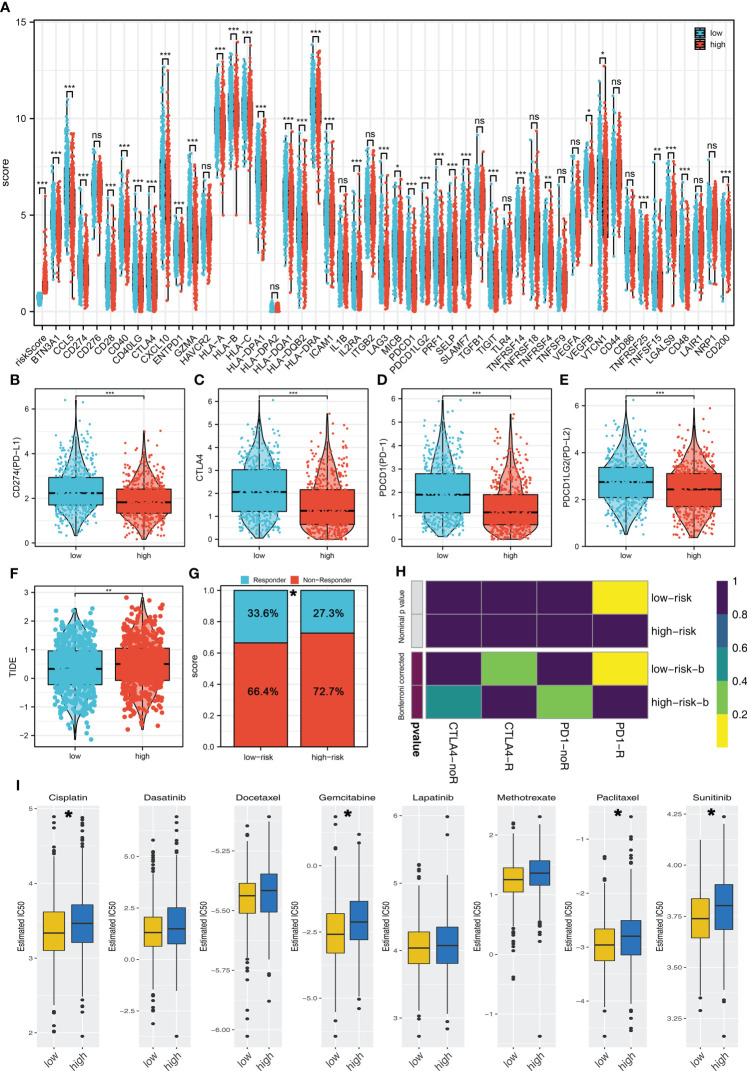
Riskscore was associated with the immunotherapy and chemotherapy sensitivity **(A)** The expression difference of immune checkpoints in high- and low-risk groups. **(B–E)** PD-L1, CTLA-4, PD-1, and PD-L2 were all highly expressed in the low-risk group. **(F)** The TIDE score in high- and low-risk groups. **(G)** Low-risk patients have a higher percentage of immunotherapy responders. **(H)** Submap algorithm indicated that the low-risk patients might be more sensitive to PD-1 therapy. **(I)** GDSC analysis showed that low-risk patients might be more sensitive to cisplatin, gemcitabine, paclitaxel, and sunitinib. *P < 0.05; **P < 0.01; ***P < 0.001.

Recently, immunotherapy has made great progress in BC. We further explored the expression difference of important immune checkpoints in high- and low-risk groups, and the result showed that multiple immune checkpoints were differentially expressed in high- and low-risk groups (**Figure 6A**). More importantly, PD-L1, CTLA-4, PD-1, and PD-L2 were all highly expressed in the low-risk group (**Figures 6B–E**). TIDE analysis indicated that the low-risk patients had a lower TIDE score, indicating that the low-risk patients might be more sensitive to immunotherapy (**Figure 6F**). Also, a higher percentage of immunotherapy responders was observed in low-risk groups (**Figure 6G**). The submap algorithm showed that the low-risk patients might be more sensitive to PD-1 therapy (**Figure 6H**). Moreover, we found that the low-risk patients might be more sensitive to cisplatin, gemcitabine, paclitaxel, and sunitinib (**Figure 6I**).

### Identification of the genes associated with the riskscore

**Figure 7:**
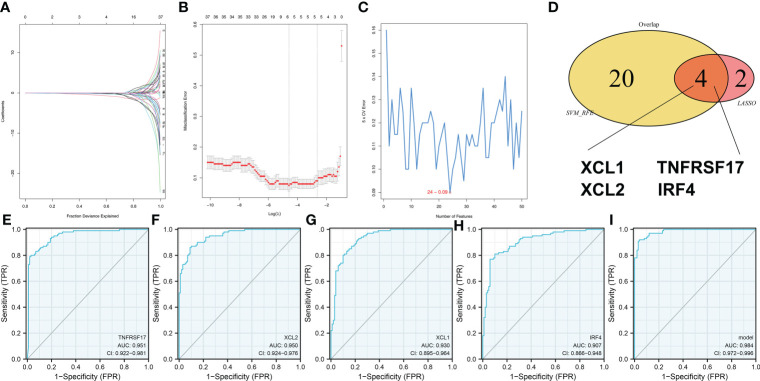
Identification of the characteristic gene of our model **(A, B)** LASSO logistic regression was used to identify the characteristic genes. **(C)** The SVM-RFE algorithm was used to identify the characteristic genes. **(D)** LASSO logistic regression and SVM-RFE intersected four genes, including *XCL1*, *XCL2*, *TNFRSF17*, and *IRF4*. **(E–H)** The ROC curve was performed to evaluate the prediction efficiency of the characteristic gene in risk group. **(I)** Logistics regression was performed to calculate the combined score, which had excellent predictive power in patients’ risk group classifications.

Next, we try to identify the characteristic genes of the risk group through two machine learning algorithms, LASSO logistic regression (**Figures 7A, B**) and SVM-RFE algorithms (**Figure 7C**). The characteristic genes identified by LASSO logistic regression and SVM-RFE intersected four genes, namely, *XCL1*, *XCL2*, *TNFRSF17*, and *IRF4* (**Figure 7D**). ROC curve was performed to evaluate the prediction efficiency of these genes in risk group. The result showed that these four genes have great prediction ability of patient risk group (**Figures 7E–H**, *TNFRSF17*, AUC = 0.951; *XCL2*, AUC = 0.950; *XCL1*, AUC = 0.930; *IRF4*, AUC = 0.907). Based on the logistic regression, a combined score was calculated with the formula “6.6356 + −2.1084 * *TNFRSF17* + −2.3712 * *XCL2* + −2.2532 * *XCL1* + 0.9918 * *IRF4*”. The ROC curve showed that the combined score had an excellent predictive power in patient risk group classifications (**Figure 7I**, AUC = 0.984).

## Discussion

BC is still a major public health problem worldwide, and its incidence has been increasing (22). For most BC patients, surgery combined with chemotherapy can lead to a good prognosis. However, a substantial proportion of BC patients have a poor prognosis due to disease recurrence and metastasis. Tumor immune microenvironment is a key factor affecting the progression of BC, and the deep exploration of it may help decipher future therapeutic targets.

In this study, 53 immune terms were quantified using the ssGSEA algorithm. Then, a prognosis model based on HER2_Immune_PCA, IL12_score, IL13_score, IL4_score, and IR7_score was established, which showed great prediction efficiency in patients’ OS. Meanwhile, univariate and multivariate analysis showed that our model is an independent risk feature for BC patients. Then, a nomogram plot was established based on the riskscore and clinical features for a better clinical application. Next, we performed clinical correlation and pathway enrichment analysis to explore the underlying mechanism of prognosis difference between high- and low-risk patients. Moreover, we found that the patients in the low-risk group might be more sensitive to the PD-1 immunotherapy, cisplatin, gemcitabine, paclitaxel, and sunitinib. Finally, the top 10 differentially expressed genes were identified, including *XCL1*, *XCL2*, *TNFRSF17*, and *IRF4*, which could contribute to risk group classification.

Tumor biology is influenced by the tumor microenvironment, especially the immune microenvironment (23). The crosstalk of immune cell, cytokine, and immune status might significantly affect tumor progression. For illustration, in the tumor microenvironment, the M1 macrophages could be activated by interferon (IFN)-γ with lipopolysaccharides and M2 macrophages could be activated by interleukin-4, which were involved in the tumor progression and metabolic reprogramming (24). In our study, we found that HER2_Immune_PCA, IL12_score, IL13_score, IL4_score, and IR7_score were significantly correlated with BC prognosis. HER2 is a classic biomarker that could indicate the molecular subtype and treatment choice of BC (25). In most cases, the HER2+ BC was considered the most dangerous subtype (25). The Interleukin family plays an important effect in BC development (26). *IL12* is an active 74-kDa heterodimer composed of an α and a β chain (27). The researchers found that IL12 could induce a Th1 response and make tumor cells exposed to the cytotoxic activity of phagocytic and NK cells, acting as an anti-tumor factor (28). Furthermore, Rahal et al. found that blocking IL4- and IL13-mediated phosphorylation of *STAT6* (Tyr641) could decrease M2 polarization of macrophages and protects against radioresistance of inflammatory BC (29).

Pathway enrichment analysis showed that the pathways of oxidative phosphorylation, E2F targets, hedgehog signaling, adipogenesis, DNA repair, glycolysis, heme metabolism, and mTORC1 signaling were activated in the high-risk group. Lee et al. found that *MYC* and *MCL1* could cooperatively promote chemotherapy-resistant BC stem cells by regulating mitochondrial oxidative phosphorylation (30). Moreover, Ramchandani et al. revealed that copper depletion could modulate mitochondrial oxidative phosphorylation to impair triple-negative BC metastasis (31). Hedgehog (Hh) signaling is crucial for embryonic development, tissue regeneration, and stem cell regeneration, which also participated in the BC progression process (32). Genomic instability is a hallmark of cancer. Germline or somatic DNA repair deficiencies in cancer could facilitate BC progression (33). Moreover, glycolysis could serve as a possible target in tumor therapies (34). Chen et al. found that extracellular vesicle-packaged HIF-1α-stabilizing lncRNA from tumor-associated macrophages could regulate aerobic glycolysis of BC cells (35).

Immunotherapy is revolutionizing the management of solid tumors (36). In BC, immunotherapy is still at the preclinical stage. Tremelimumab and ipilimumab, two CTLA-4 antagonists, have been tested in small BC trials with some evidence of immune modulation (37). In addition, the growing body of evidence suggests that PD-1/PD-L1 antagonists may induce durable clinical responses in metastatic TNBC patients (36). Chemotherapy was one of the most important treatment options for cancer and many trials are exploring the feasibility of adding chemotherapy to PD-1/PD-L1 therapy (38). Our result showed that the patients in the low-risk group might be more sensitive to PD-1 therapy, cisplatin, gemcitabine, paclitaxel, and sunitinib. Also, four genes, *XCL1*, *XCL2*, *TNFRSF17*, and *IRF4*, were identified for risk group classification. In the clinical setting, detection of the expression level of these genes with breast biopsy might indicate the prognosis and therapy option of patients.

In all, our study quantified the immune microenvironment of BC and established a prognosis model that could effectively predict the prognosis. Also, the underlying biological mechanism and drug sensitivity differences were explored between the high- and low-risk groups. However, some limitations should be noticed. Firstly, the population used for analysis was predominantly Western populations, which might lead to underlying race bias. This might affect the application of our model in other races. Secondly, the distant metastasis information of our samples was mostly unknown, which might affect the clinical application of our model. Thirdly, the result of our study is based on mRNA level, but not protein level. Therefore, it may not fully represent the real situation of patients.

## Data availability statement

Publicly available datasets were analyzed in this study. This data can be found here: https://portal.gdc.cancer.gov/; https://www.ncbi.nlm.nih.gov/gds/.

## Author contributions

YD and JW collected the data and performed all analysis. YD and ZH wrote the manuscript. All authors contributed to the article and approved the submitted version.

## Funding

The project was funded by the School Foundation of Chengdu Medical College (Grant No. CYZYB21-05), the Project of Chengdu Medical Research (Grant No. 2021015) and the Xiangtan Medical Research Project (No. 2020xtyx-3).

## Conflict of interest

The authors declare that the research was conducted in the absence of any commercial or financial relationships that could be construed as a potential conflict of interest.

## Publisher’s note

All claims expressed in this article are solely those of the authors and do not necessarily represent those of their affiliated organizations, or those of the publisher, the editors and the reviewers. Any product that may be evaluated in this article, or claim that may be made by its manufacturer, is not guaranteed or endorsed by the publisher.

## Supplementary material

Supplementary Figure 1 The flowchart of the whole study.

Supplementary Figure 2 Exploration of the underlying biological difference between different age and risk patients. **(A)** GSEA was performed in ≤60- and >60-year-old patients. **(B)** GO and KEGG analysis of high- and low-risk patients.
