# Supplementary figures and images for: Exploration of the immune microenvironment of breast cancer in large population cohorts
Source: Front Endocrinol (Lausanne). 2022 Aug 15;13:955630. doi: 10.3389/fendo.2022.955630 (PMC9421148; doi:10.3389/fendo.2022.955630)

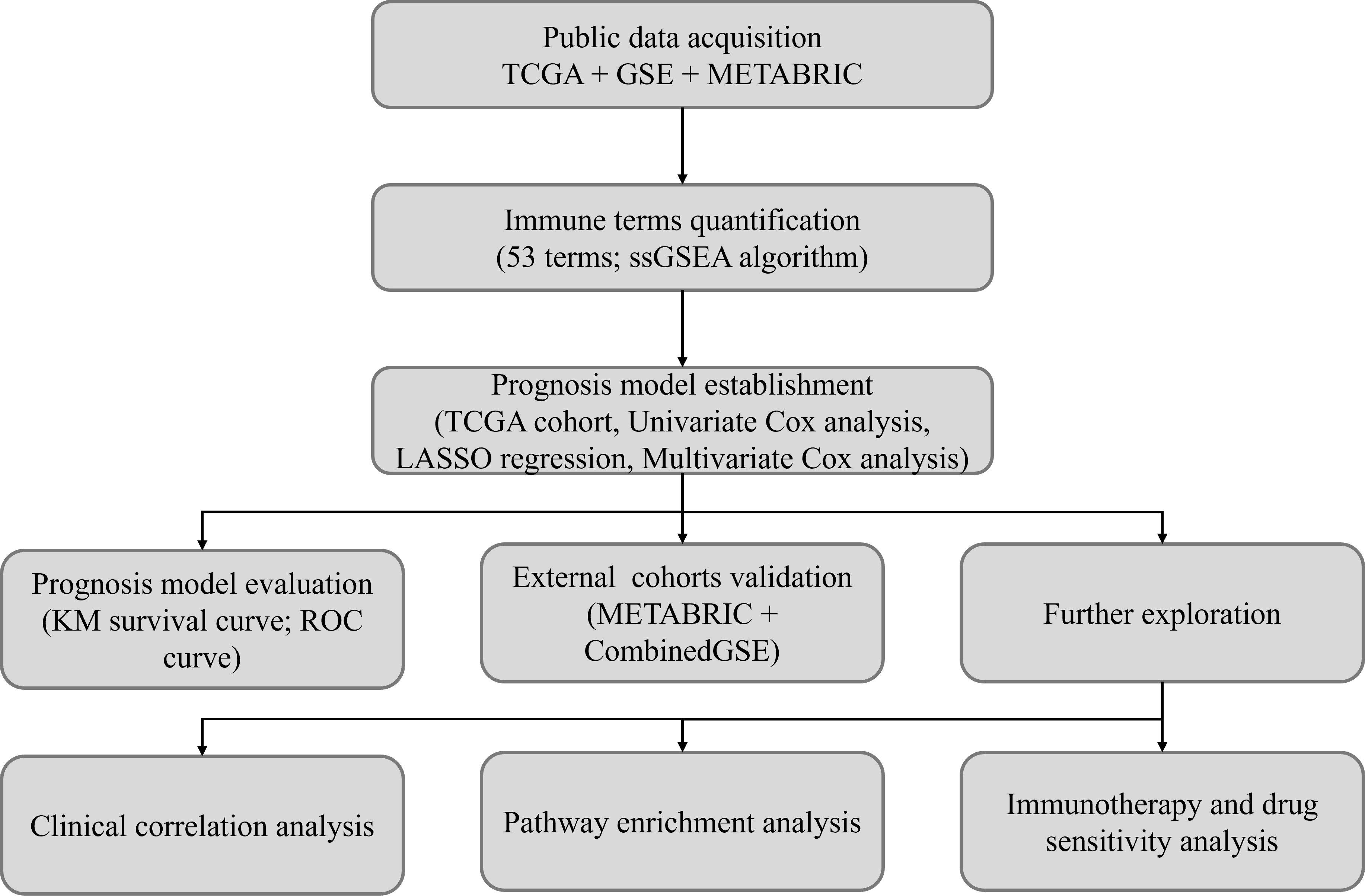

Supplement: Supplementary Figure 1 — The flowchart of the whole study. [file Image_1.tif]

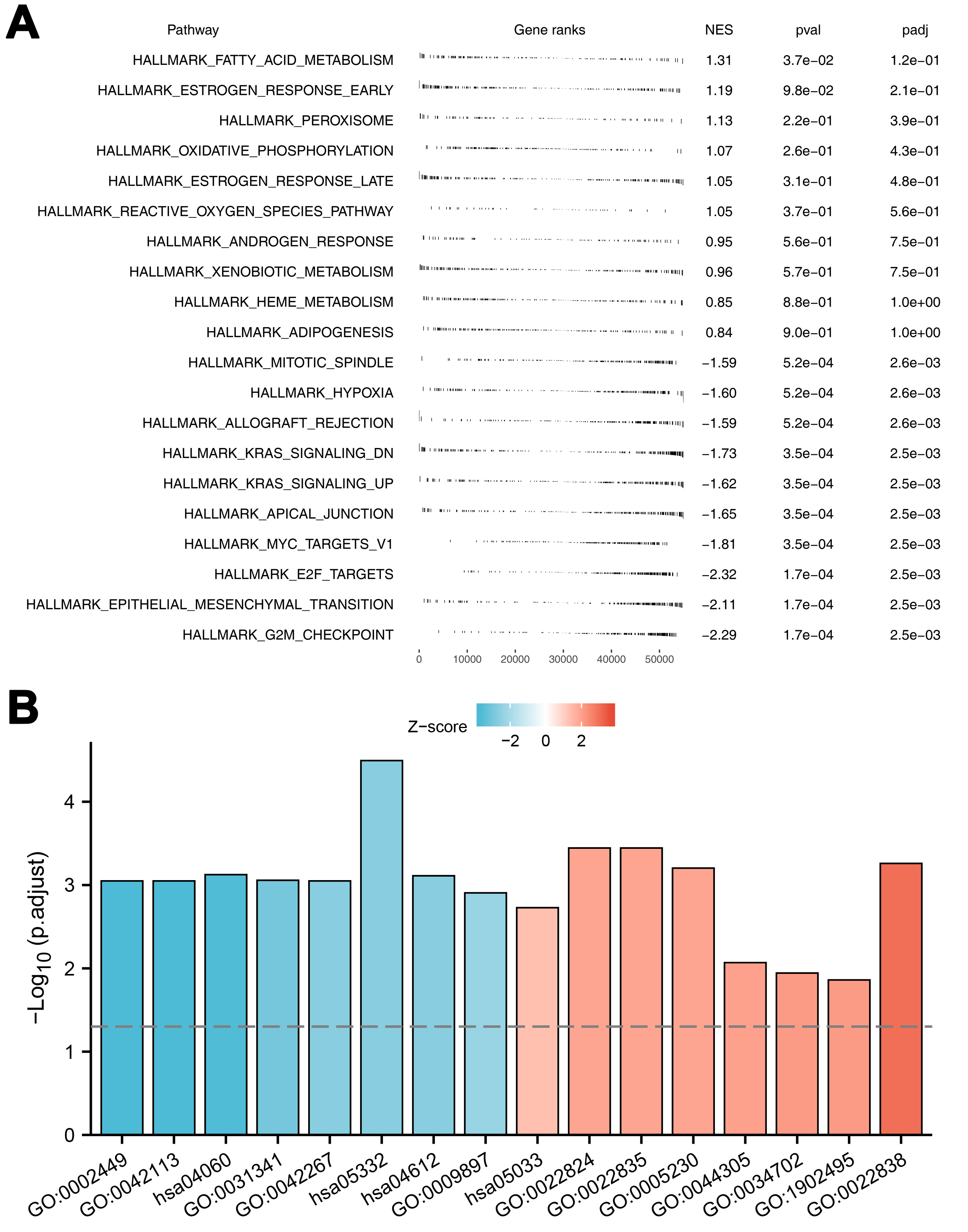

Supplement: Supplementary Figure 2 — Exploration of the underlying biological difference between different age and risk patients. (A) GSEA was performed in ≤60- and >60-year-old patients. (B) GO and KEGG analysis of high- and low-risk patients. [file Image_2.tif]
